# Pathological Features in 100 Deceased Patients With COVID-19 in Correlation With Clinical and Laboratory Data

**DOI:** 10.3389/pore.2021.1609900

**Published:** 2021-08-06

**Authors:** L. M. Mikhaleva, A. L. Cherniaev, M. V. Samsonova, O. V. Zayratyants, L. V. Kakturskiy, O. A. Vasyukova, A. E. Birukov, A. S. Kontorshchikov, A. V. Sorokina, M. Y. Sinelnikov

**Affiliations:** Research Institute of Human Morphology, Moscow, Russia

**Keywords:** histological subtype, COVID-19, pathological features, large cohort, morphology

## Abstract

**Background:** Autopsies on COVID-19 deceased patients have many limitations due to necessary epidemiologic and preventative measures. The ongoing pandemic has caused a significant strain on healthcare systems and is being extensively studied around the world. Clinical data does not always corelate with post-mortem findings. The goal of our study was to find pathognomonic factors associated with COVID-19 mortality in 100 post-mortem full body autopsies.

**Materials and Methods:** Following necessary safety protocol, we performed 100 autopsies on patients who were diagnosed with COVID-19 related death. The macroscopic and microscopic pathologies were evaluated along with clinical and laboratory findings.

**Results:** Extensive coagulopathic changes are seen throughout the bodies of diseased patients. Diffuse alveolar damage is pathognomonic of COVID-19 viral pneumonia, and is the leading cause of lethal outcome in younger patients. Extrapulmonary pathology is predominantly seen in the liver and spleen. Intravascular thrombosis is often widespread and signs of septic shock are often present.

**Conclusion:** The described pathological manifestations of COVID-19 in deceased patients are an insight into the main mechanisms of SARS-CoV-2 associated lethal outcome. The disease bears no obvious bias in severity, but seems to be more severe in some patients, hinting at genetic or epigenetic factors at play.

## Introduction

The ongoing SARS-CoV-2 outbreak has caused significant healthcare, social and mental difficulties [1]. On March 12th, 2020 the World Health Organization announced the outbreak a pandemic. More than 180 million cases of COVID-19 have now been registered by now in the world [2]. Lethality has been officially registered at 0.1–0.5% globally [2], yet many governments have been accused of artificially reducing both infection and lethality rates through falsification of data or underreporting [3,4]. Nevertheless, the toll of the ongoing SARS-CoV-2 pandemic has already brought significant social tension, healthcare straining and overall decrease in quality of life [5–7]. The pathological characteristics of COVID-19 pulmonary changes have been previously addressed, yet most studies present a small number of cases [8,9]. Several reports of pathological characteristics of extrapulmonary manifestations have also been reported [10–12].

The goal of our study was to assess the pathological features of COVID-19-related deaths during autopsy in a substantial cohort (100 cases). Our null hypothesis was that all cases of lethal outcome have similar associations and features, according to treatment modality and patient characteristics.

## Materials and Methods

Our study included clinical, laboratory and pathology findings collected from 100 deceased COVID-19 patients. Demographic, clinical data (including available patient history and comorbidities), computerized tomography (CT) results, macroscopic and microscopic assessment of pathological changes in the entire body was performed.

COVID-19 post-mortem verification. COVID-19 infection was confirmed in all patients by post-mortem PCR-testing (polymerize-chain reaction test) of biomaterial collected from the trachea, major bronchi and lungs. PCR-testing was performed for all patients, even when a COVID-19 diagnosis was already issued prior to lethal outcome.

Safety protocol. The autopsies were performed according to World Health Organization (WHO) and the Health Ministry of Russia recommendations. Pathologists and assistants worked in disposable full-body Taiwek suits, plastic safety glasses, two pairs of disposable gloves, class FPP3 inhalers, disposable aprons, shoelaces. Two staff members were allowed to be in the dissection room at any given time (a pathologist and a dissector). Brain examination was performed after manual skull sectioning. Electric sawing was not permitted. Following autopsy and tissue extraction, all instruments underwent disinfection in an antiseptic solution, all disposable utilities were utilized.

Pathological evaluation. Excised tissue fragments were fixed in 10% neutral formalin solution using the automatic histologic processor Leica ASP 300S (Germany). Paraffin filling was then performed using the Leica EG 1160 station (Germany). A Leica RM 2125 RTS (Germany) microtome was used to provide 3–4 μm thick slices. Hematoxylin and eosin, periodic acid-Schiff (PAS), and Alcian blue staining were performed using the Leica ST 5010 (Germany). Perls staining was used for histochemical detection of iron. The following organs were evaluated in all patients: brain; trachea and bronchi; lungs; bifurcation lymph nodes; heart; kidneys; spleen; liver.

Immunohistochemistry (IHC). Histological sections also underwent IHC evaluation. The produced 3–4 μm sections were placed on super adhesive slides (Trajan T7611), after which they were placed for 2 h into a thermostat (70°С). The staining was performed using double-stage avidin-biotin-peroxidase method with antigen unmasking solutions, polyclonal and monoclonal antigen use. IHC staining was performed using the Ventana BenchMark ULTRA IHC/ISH (United States) and Leica Bond-MAX (Germany). The following antibodies were used in our study: CD31 Mouse Monoclonal Antibody (clone JC70, ready-to-use), CK5/6 Mouse Monoclonal Antibody (clone D5/16B4, ready-to-use).

Microscopic evaluation. Microscopically the specimens were analyzed using the triocular Leica DMLB (Germany) microscope with a fully-compatible Leica DFC 420 (Germany) digital camera. Final image analysis was performed by ImageScope Color M software. Calibration was performed by an object-micrometer with 0.01 mm step.

During microscopic evaluation signs of respiratory distress syndrome (exudative and proliferative phases) were noted. The following parameters were analyzed: hyaline membrane structure, fibrin deposits, interalveolar bleeding, interalveolar edema, interstitial inflammation, cytopathic effects, granulation tissue (fibroblastic tissue), thrombi presence within vasculature, infarctions, neutrophilic groups, macrophages, lymphocytes, plasma cells, and siderophages within the tissue (and localization), squamous cell metaplasia and bronchial epithelium desquamation, squamous cell metaplasia and alveolar epithelium desquamation, macrophage presence within the alveoli, signs of viral presence (*via* cytopathic effect characterized by the formation of large cells of irregular shape with enlarged nuclei and coarse-grained chromatin, distinct nucleoli, with a perinuclear “halo” effect), myxoid edematous interstitial stroma presence, signs of aspiration, tissue necrosis. Pathological findings within the cohort are represented as mean and standard deviation.

Visual evaluation of the microscopic changes of other organs and tissues was also performed to identify pathologic changes. Organs showing signs of pathological change during autopsy were examined pathologically. Clinical indications and patient history were taken into account. All organs and systems underwent primary evaluation and were included in the study if pathological changes were noted.

Statistical evaluation. The significance of differences between sub-cohorts was determined using the independent *t*-test or the nonparametric Mann-Whitney *U*-test when variables were non-normally distributed. Comorbidity rates were compared using Pearson’s chi-squared test or Fisher’s exact test. Analysis of variance was performed to evaluate intergroup differences on each parameter with appropriate data. The minimal number of cases that needed to be included in this study was calculated using power analysis. Statistical data was calculated using RStudio software, version February 1, 1335 (RStudio, Inc., Boston, MA, United States). Results are presented as means ± standard deviation or as numbers and percentages, and statistical significance was set at *p*-values < 0.05.

## Results

### Patient Characteristics

**FIGURE 1:**
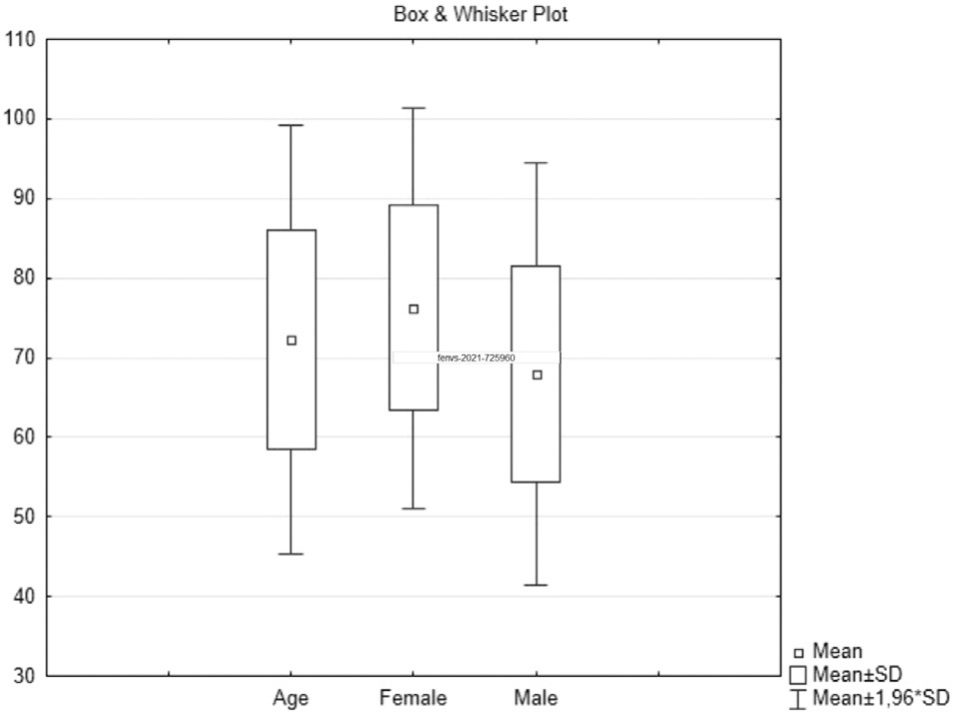
COVID-19 related autopsy distribution by sex and age, mean ± standard deviation.

A total of 100 autopsies were performed (53 females, 47 males). The mean age of the deceased patients was 70.8 years (range: 45–95 years). Mean female age was 75.5 (range: 58–95 years). Mean male age was 67.5 (range: 45–92 years) (Figure 1). The mean duration of the disease until lethal outcome was 13.4 days (range: 2–48 days). The mean hospitalization duration was 7.48 days (range: 5–25 days).

**FIGURE 2:**
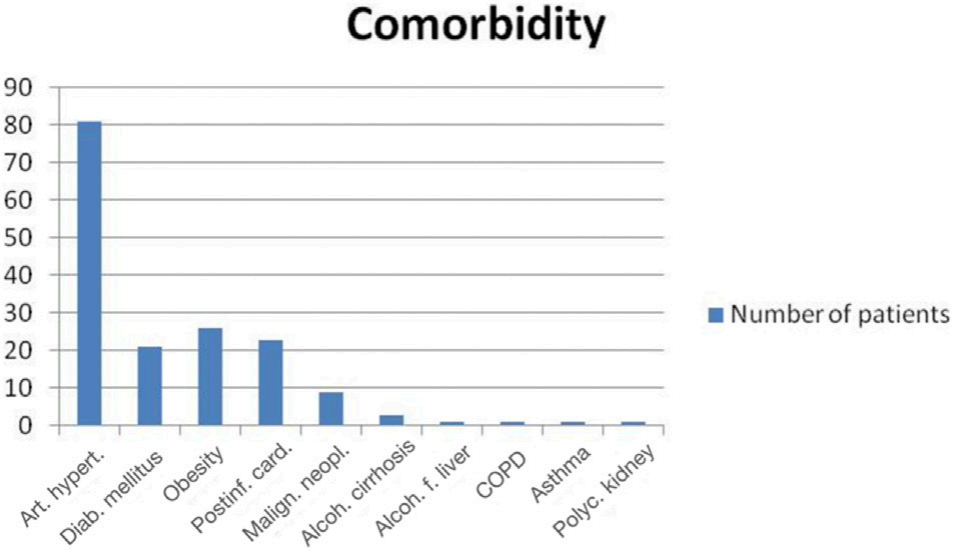
Comorbidities in COVID-19 deceased patients [Art. hypert.—arterial hypertension (81/100), Diab. Mellitus—diabetes mellitus (21/100), Obesity (26/100), Postinf. card.–postinfarction cardiosclerosis (23/100), Malign. Neopl.—malignant neoplasms (8/100), Alcoh. cirrhosis—alcoholic cirrhosis of liver (3/100), Alcoh. f. liver—alcoholic fatty liver (1/100), COPD—chronic obstructive pulmonary disease (1/100), Asthma (1/100), Polyc. kidney—polycystic kidney (1/100)].

Most of the deceased patients had aggravating conditions (Figure 2). Hypertensions was the most common comorbidity. 48% of patients had more than one comorbidity. Eight patients presented with malignancies: lung, breast, colon cancers, B-cell and T-cell lymphoma, cutaneous plasmacytoma (one case each), and chronic lymphocytic leukemia (two patients). Alcohol related hepatic micronodular cirrhosis was observed in three patients, one of which was diagnosed with the hepatocellular carcinoma. Hepatic steatosis without cirrhosis was noted once. Two deceased patients were diagnosed with COPD and bronchial asthma, one patient had a congenital polycystic kidney disease, and another one suffered from systemic amyloidosis with cardiac involvement.

**FIGURE 3:**
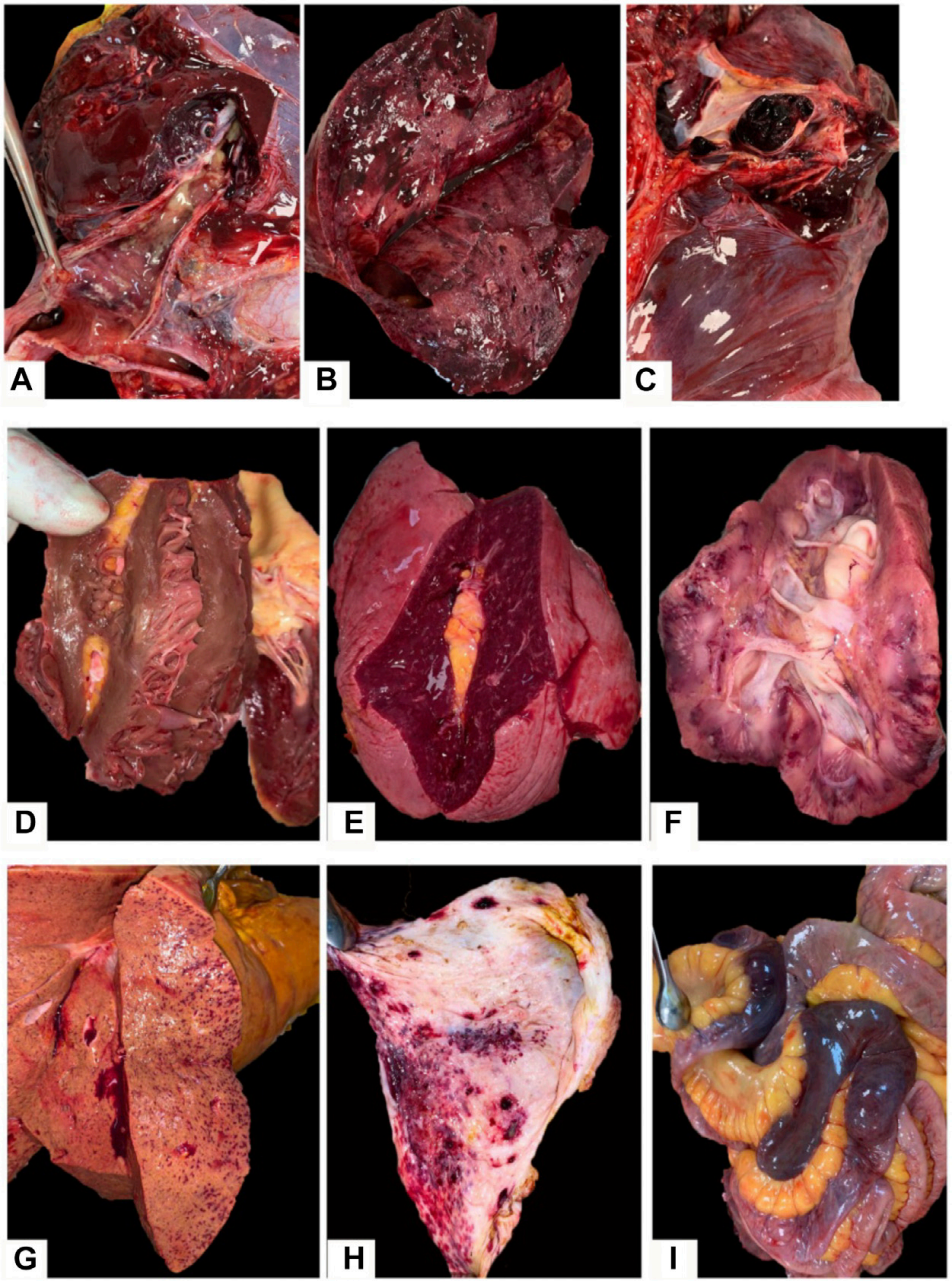
Macroscopic findings in COVID-19 patients: **(A–C)**–pulmonary surface with a dark-red or dark-cherry color, volume increase and airless tissue, with a rubber density, dark-red or brown-red in color with pulmonary emboli detected in lung arteries lumens; **(D)**–left ventrical hypertrophia with pale ischemic metabolic focus; **(E)**—enlarged spleen with average pulp scraping; **(F)**—“shock” kidney with pale cortex and congested medullae with hemorrhages; **(G)**—liver tissue demonstrating fatty degeneration and congested vessels with focal hemorrhages; **(H)**–hemorrhages in the gastric mucosa in a COVID-19 patient complicated by DIC; **(I)**—segmental gangrene of the intestine in a COVID-19 patient complicated by DIC.

Disseminated intravascular coagulation (DIC) syndrome was seen in 29 cases. It presented with serosal and mucosal hemorrhages (in 96% of cases) (Figure 3H), uncoagulated blood in the blood vessels and heart (in 79.3% of patients) and acute erosions within the gastrointestinal (GI) tract (in 44.8% of patients). Pulmonary thrombosis was found in 44.8% of patients effected by the DIC syndrome (13/29), Myocardial ischemic pathology was observed in 17.2% of patients (5/29), Intestinal gangrene was seen in 10.3% of patients (3/29) (Figure 3I). Gastrointestinal bleeding was noted in 10.3% of DIC cases (3/29).

### Pulmonary Findings

Macroscopically, the pulmonary tissues of deceased patients showed signs of acute respiratory damage in the form of “shock lung” (varnish pulmonary surface with a dark-red or dark-cherry color, volume increase (from 1,100 to 2,860 g, mean volume is 1,659 g), and the pulmonary consolidation. Lung surface was airless, with a rubber density, dark-red or brown-red in color (Figures 3A,B). Pulmonary embolism was seen in 19 patients (Figures 3A,C). 31 patients showed signs of viral-bacterial pneumonia, and three patients developed mixed viral-bacterial-fungal pneumonia, confirmed by *in-vivo* microbiology assessment, mucus analysis and postmortem diagnosis.

Microscopic signs of diffuse alveolar damage (DAD) was seen as the morphologic equivalent of viral pneumonia, the signs of which were analyzed by a modification of T. Mauad et al. algorithm for pulmonary changes in influenza A (H1N1) patients (Table 1) [12].

**TABLE 1:** Microscopic changes in lungs.

Microscopic findings	Number of patients with the finding (N)
Hyaline membranes	75
Fibrin within the alveoli	54
Interalveolar hemorrhages	75
Siderophages within the alveoli	43
Interalveolar edema	61
Interstitial inflammation	38
Cytopathic effect	55
Granulation tissue	28
Pulmonary infarction	31
Arterial thrombi	67
Vein thrombi	48
Neutrophils in the bronchi/bronchioles/alveoli	29
Bronchial epithelium metaplasia	24
Bronchial epithelium desquamation	67
Alveolar epithelium metaplasia	37
Alveolar epithelium desquamation	67
Alveolar macrophages	56
Intercellular viral particles	0
Interstitial myxoid edematous stroma (alveolar walls, perivascular)	19
Aspiration	1
Plasma cells, lymphocytes within alveoli	43
Siderophages within the bronchi	12

**FIGURE 4:**
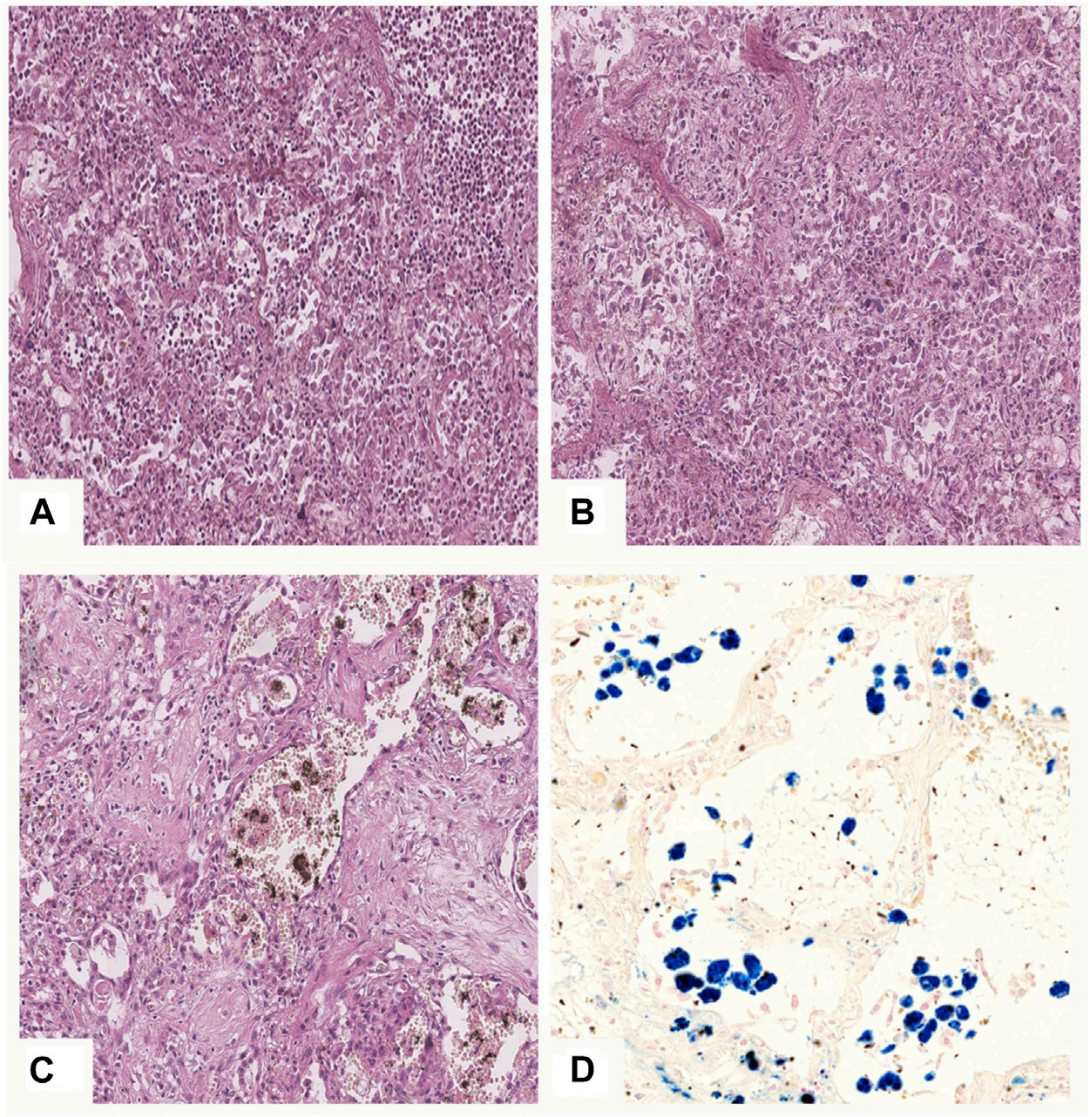
Pulmonary microscopic findings in COVID-19 patients. **(A)**—DAD phase 1: hyaline membranes lining the alveoli, desquamation of alveolocytes, focus of leukocyte infiltration (upper right corner); **(B)**—DAD mixed phase—alongside hyaline membranes there are fibroblasts and granulation tissue present; **(C)**—DAD phase 2: fibrous tissue with single alveolar lumens detected, erythrocytes and hemosyderophages in the alveolar lumens; **(D)**—iron deposits in hemosyderophages in alveolar lumens. **(A–C)**—H&E stain, ×20; **(D)**—Perls’ stain, ×20.

In microscopic examination of the lungs pulmonary edema was found in 61 cases, hyaline membranes within alveolar walls and lumens was seen in 75 cases, alveolar epithelial plast-like desquamation in 67 patients. The cytopathic effect is seen as large alveolocytes with rubber chromatic nuclei and nucleoli presence, occasionally with a perinuclear “halo” and round-shape intracytoplasmic inclusions with signs of interstitial inflammation and occasional megakaryocytes: this phenomenon was seen in 55 cases. Among typical DAD findings vascular injuries were present: interalveolar capillary, vein and venular dilatation, interalveolar hemorrhages and arteriospasm were observed in 75 cases, although its severity varied from microscopic to vast pulmonary infarctions (31 cases) (Figure 4).

In the brain, both in the cortical and in the medulla and in the region of the basal nuclei, pronounced pericellular and perivascular edema, diapedesis and focal hemorrhages with saturation of adjacent brain tissue with blood were found. It is believed that violations of permeability of the blood-brain barrier and associated neural tissue damage are the result of the cytokine storm.

When examining the urogenital tract, with the exception of the kidneys, no specific changes were noted. In isolated cases, focal or confluent hemorrhages were revealed in the mucous membrane of the renal pelvis and ureters, as well as in the mucous membrane of the bladder, associated with systemic manifestations of DIC syndrome.

Edema and focal hemorrhages were found in the parotid glands, however, we did not find any changes in other salivary glands. Purulent tracheobronchitis was found in 34% of cases, hemorrhagic tracheobronchitis–in 10%, and mixed, purulent-hemorrhagic tracheobronchitis in 4 cases. These changes were most often associated with tracheal intubation and prolonged mechanical ventilation.

Red blood cell stasis with arterial fibrinous and organized thrombi were identified in 67 cases, the same findings were observed in veins in 48 cases. This is typical of fibrin cloths. The presence of alveolar siderophages, and siderophages were identified in bronchi and terminal bronchioles in 43 cases. Interalveolar and interstitial plasma cell and lymphocyte infiltration was found in 43 cases, although its severity varied. In 38 cases interstitial inflammation of the interalveolar septae was seen. 19 cases demonstrated a perivascular myxoid edematous stroma and similar stromal changes within the interalveolar septae. Terminal bronchiolar epithelial (*n* = 24) and alveolar (*n* = 37) squamous cell metaplasia, and its rare desquamation into the bronchi (*n* = 67) and alveoli in the form of plasts (*n* = 67) was common.

**FIGURE 5:**
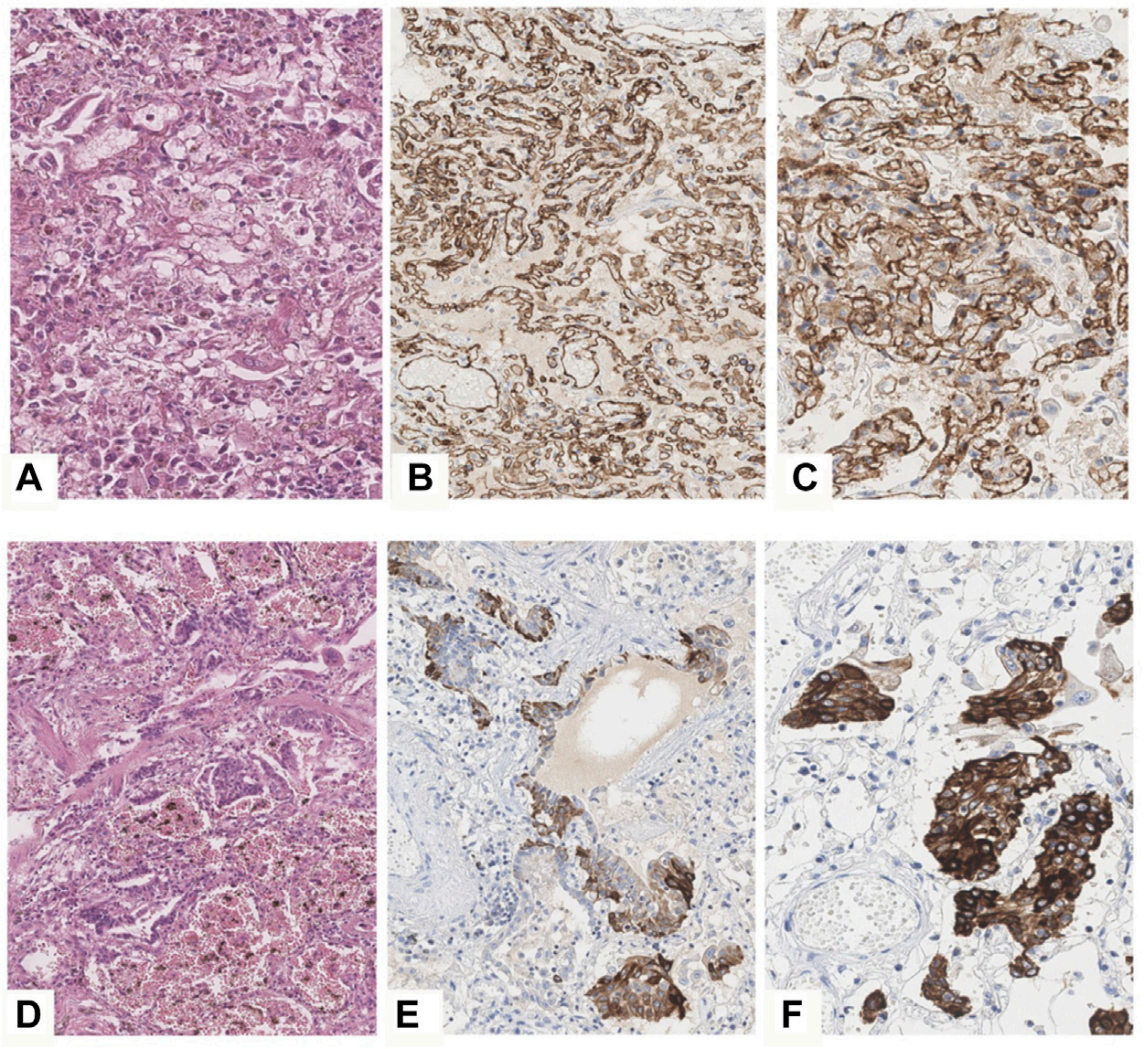
DAD phase 2 in COVID-19 patiens. **(A–C)**—vascular proliferation in lungs. **(А)**—H&E, ×40, **(B,C)**—IHC with the CD31 marker, B×20, **(C)**—×40. **(D–F)**—squamous cell metaplasia of an alveolar epithelium and bronchi. **D**—H&E, ×20, **(E,F)**—IHC staining with the CK5-6 marker, **(E)**—×20, **(F)**—×40.

Vascular proliferation was identified in the form of multiple closely related thin-walled capillaries, confirmed by IHC staining with a CD31 marker (Figures 5A–C). Squamous cell metaplasia was characterized by a highly intensive membranous staining of epithelial cells in immunohistochemical processing by CK5-6 (Figures 5D–F). Based on morphologic findings, exudative (early) phase DAD was diagnosed in 45 cases. Proliferative (late) phase of DAD characterized by the fibrin deposits of different maturity within the alveoli and single terminal and respiratory bronchioles, also by polypoid fibroblastic tissue (granulation tissue) within the terminal bronchioles and alveoli was diagnosed in 45 cases. The exudative and productive phases of diffuse alveolar damage (mixed DAD phase) with signs of local interalveolar edema, hyaline membranes, desquamated alveolar epithelium combined with fibrin, alveolar fibroblastic component, alveolar and bronchial squamous cell metaplasia were found in 36 autopsy cases. In 31 cases, microscopic findings of bronchopneumonia, and in 3 cases viral-bacterial-mycotic pneumonia were identified, and it was proven by microbiological evaluation. In 1 case only a bacterial aspiration bronchopneumonia with no DAD criteria was identified, despite positive PCR testing.

### Extrapulmonary Findings

**FIGURE 6:**
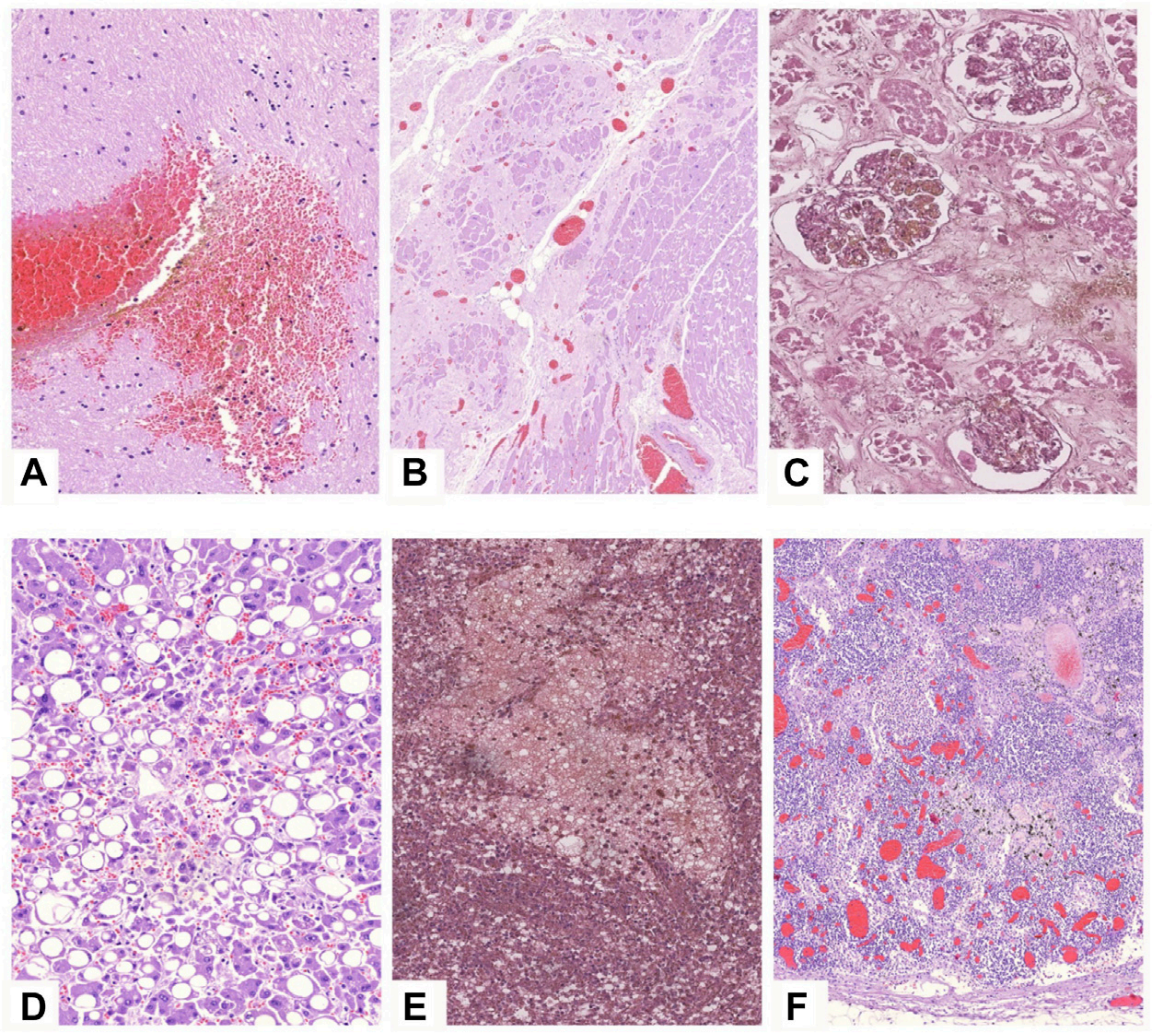
Microscopic findings in other organs in COVID-19 patients. H&E stain. **(A)**—brain tissue demonstrating focal hemorrhage and pericellular edema, ×20; **(B)**—miocard sample with large postinfarction cardiosclerosis area, congested vessels and contracted cardiomyocytes, ×5; **(C)**—kidney specimen showing massive necrosis of tubular epithelium, ×20; **(D)**—steatosis of hepatocytes, congested vessels, ×20; **(E)**—focal hemorrhage in the spleen, ×20; **(F)**—reactive lymphadenitis with angiomatosis of bifurcation lymphnodes.

Hemmoragic findings in the brain were found in seven cases (Figure 6A). Hepatomegaly and splenomegaly of ranging severity were identified in all COVID-19 autopsies. Heart weight varied from 250 to 700 g (mean = 452 g). The ventricular index in 81% of cases was less than 0.4, which testifies arterial hypertension. Postinfarction cardiosclerosis was observed in 23 patients (Figure 6B). Myocardial metabolic disorders were identified in 6 cases, five of which—in patients with DIC syndrome, which was interpreted as hemopoietic complications of COVID-19 infection in case an atherosclerotic plaque was absent within the coronary arteries (Figure 3D). Ischemic and hemorrhagic infarctions of the kidneys were identified in 2 cases, one of which was associated with DIC syndrome. “Shock kidneys” were noted in five autopsies, which demonstrated signs of acute renal failure (Figure 3F). Microscopically along with an arteriolonephrosclerosis (common finding in hypertensive patients), partial glomerular collapse, significant granular tubular epithelial dystrophy, renal tubular eosinophilic masses, tubular and interstitial necrosis and edema were commonly identified (Figure 6C).

The spleen varied in weight being 50–530 g (mean = 176 g). Splenic hyperplasia (over 190 g) with an notable pulp scraping was detected in 31 cases (Figure 3E). Increased splenic weight was more pronounced in patients with sings of septic shock. Microscopically a follicular reduction, splenic pulp unification with lymphocytes and multiple necrotic foci, and hemorrhages was noticed (Figure 6E). Focal hemorrphages as well as angiomatosis were also observed in bifurcation lymphnodes (Figure 6F). The livers had signs of droplet-like, middle-size and large droplet-like steatosis of ranging severity in all cases along with petechial hemorrhages (Figures 3G, 6D).

Clinical correlation. In order to find significant difference between patient groups, we decided to evaluate the most common comorbid conditions in the deceased patients. These included arterial hypertension, obesity, ischemic heart disease and diabetes. The patients were categorized into eight groups according to combination of these comorbid conditions (Table 2). Analysis of variance showed that the mean respiration rate, C-reactive protein levels, heart mass, lung mass and CT severity grades were the only variables with significant differences. Specifically, patients with all four comorbidities had a significantly higher mean respiration rate. C-reactive protein levels were significantly greater in patients with comorbidities compared to those without. Interestingly, lung mass was greater in the group of patients with arterial hypertension, obesity and ischemic heart disease, compared to those without comorbid conditions. Pulmonary findings did not vary significantly in patients according to clinical findings (Table 3).

**TABLE 2:** Comparison of clinical data between patients with common comorbid conditions (AH, arterial hypertension; OB, obesity; DM, diabetes mellitus; ICD, ischemic heart disease).

Cat	A	B	C	D	E	F	G	H	
Comorbidity	Arterial hypertension only (*n* = 33)	Arterial hypertension and Obesity (*n* = 8)	AH + OB + DM + ICD (*n* = 2)	AH + OB + DM (*n* = 9)	AH + DM (*n* = 7)	AH + ICD (*n* = 17)	AH + OB + ICD (*n* = 3)	None (*n* = 15)	*p*-value (ANOVA)
Age	71.73 ± 14.14	73.38 ± 16.28	68.50 ± 4.95	71.67 ± 13.37	72.71 ± 11.53	72.53 ± 13.63	86.67 ± 6.43	69.07 ± 12.81	0.656
Time from hospitalization to death (days)	8.12 ± 5.07	6.13 ± 7.90	12.50 ± 4.95	6.36 ± 3.38	7.57 ± 4.86	6.94 ± 4.44	13.33 ± 10.69	7.33 ± 4.69	0.430
Disease duration (symptom onset to death)	14.46 ± 6.19	11.38 ± 10.14	14.50 ± 6.36	11.11 ± 4.37	15.29 ± 6.32	14.06 ± 3.19	20.67 ± 23.67	11.20 ± 4.60	0.321
Time on ventilation	5.73 ± 5.40	6.17 ± 9.37	10.50 ± 6..36	5.27 ± 2.84	7.44 ± 5.01	5.31 ± 3.52	11.67 ± 11.93	7.08 ± 3.96	0.535
Patient mean respiration rate (breaths/min)	20.85 ± 2.64	21.00 ± 2.53	32.50 ± 17.58	21.29 ± 3.20	23.40 ± 5.94	21.91 ± 4.01	22.67 ± 3.06	22.86 ± 4.63	0.0189* C vs A,B,D,F,H
Maximum C-reactive protein (mg/L)	245.70 ± 96.23	152.52 ± 89.07	198.20 ± 4.38	141.95 ± 57.98	237.90 ± 73.02	134.43 ± 94.12	141.50 ± 61.52	59.54 ± 51.57	<0.0001* A vs D,F,H E vs H
SpO^2^ on admission (%)	87.23 ± 10.35	87.33 ± 6.19	78.00 ± 16.97	86.67 ± 21.11	84.50 ± 9.15	85.00 ± 13.28	84.00 ± 8.49	90.40 ± 6.11	0.850
Mean body temperature (C^o^)	38.27 ± 0.97	37.86 ± 0.77	37.75 ± 0.35	37.45 ± 1.88	38.46 ± 0.55	38.26 ± 0.63	38.75 ± 1.48	38.50 ± 0.86	0.216
Maximum lymphocyte content (%)	13.41 ± 12.77	10.42 ± 3.36	3.50 ± 2.40	13.85 ± 7.57	13.00 ± 8.34	15.40 ± 18.38	8.6 ± 3.39	10.57 ± 3.00	0.828
Heart mass (g)	471.27 ± 96.97	431.00 ± 111.17	383.00 ± 45.25	440.89 ± 89.66	464.29 ± 84.63	406.18 ± 91.42	313.33 ± 77.67	483.33 ± 90.05	0.0443*
Spleen mass (g)	178.33 ± 96.88	163.63 ± 95.74	134.00 ± 50.91	252.33 ± 138.29	138.57 ± 37.61	169.06 ± 82.47	120.00 ± 17.32	160.00 ± 61.96	0.202
Lung mass	1729.39 ± 465.11	1,620.63 ± 300.01	1962.50 ± 901.56	1,641.11 ± 425.89	1,467.14 ± 290.62	1,617.64 ± 264.21	2,283.33 ± 325.32	1,499.33 ± 270.49	0.0484*
G vs H									
CT severity (grade 1–5)	3.33 ± 0.58	2.50 ± 2.12	3.50 ± 0.71	3.14 ± 0.90	3.14 ± 0.89	3.75 ± 0.50	3.67 ± 0.58	3.5 ± 0.71	0.0875 B vs. F (0.0263*)

Bold values: statistically significant findings at *p* < 0.05.

**TABLE 3:** Pulmonary findings (AH, arterial hypertension; OB, obesity; DM, diabetes mellitus; ICD, ischemic heart disease).

Comorbidity	Arterial hypertension only (*n* = 33)	Arterial hypertension and obesity (*n* = 8)	AH + OB + DM + ICD (*n* = 2)	AH + OB + DM (*n* = 9)	AH + DM (*n* = 7)	AH + ICD (*n* = 17)	AH + OB + ICD (*n* = 3)	None (*n* = 15)
DAP phase 1 (*n*, %)	16 (48.48%)	4 (50.00%)	0 (0.00%)	2 (22.22%)	2 (28.57%)	10 (58.82%)	2 (66.67%)	8 (53.33%)
DAP phase 2 (*n*, %)	6 (18.18%)	2 (25.00%)	1 (50.00%)	3 (33.33%)	3 (42.86%)	2 (11.76%)	0 (0.00%)	0 (0.00%)
DAP phase 1–2 (*n*, %)	11 (33.33%)	2 (25.00%)	1 (50.00%)	4 (44.44%)	2 (28.57%)	5 (29.41%)	1 (33.33%)	7 (46.67%)
Hyaline membrane presence	25 (75.76%)	6 (75.00%)	1 (50.00%)	4 (44.44%)	4 (57.14%)	14 (82.35%)	3 (100%)	15 (100%)
Fibrin in alveoli	17 (51.52%)	3 (37.50%)	2 (100%)	7 (77.78%)	5 (71.43%)	6 (35.29%)	2 (66.67%)	7 (46.67%)
Erythrocytes in alveoli	21 (63.64%)	5 (62.50%)	1 (50.00%)	8 (88.89%)	7 (100%)	16 (94.12%)	3 (100%)	11 (73.33%)
Siderophages in alveoli	9 (27.27%)	3 (37.50%)	1 (50.00%)	6 (66.67%)	5 (71.43%)	9 (52.94%)	0 (0.00%)	6 (40.00%)
Interalveolar oedema	22 (66.67%)	4 (50.00%)	1 (50.00%)	5 (55.56%)	3 (42.86%)	10 (58.82%)	3 (100%)	12 (80.00%)
Interstitial inflammation	13 (39.39%)	3 (37.50%)	2 (100%)	4 (44.44%)	4 (57.14%)	5 (29.41%)	0 (0.00%)	4 (26.67%)
Cytopathic effect	14 (42.42%)	7 (87.50%)	0 (0.00%)	5 (55.56%)	5 (71.43%)	14 (82.35%)	0 (0.00%)	7 (46.67%)
Granulations	10 (30.30%)	3 (37.50%)	2 (100%)	3 (33.33%)	5 (71.43%)	2 (11.76%)	0 (0.00%)	1 (6.67%)
Hemorrhagic infarctions	12 (36.36%)	0 (0.00%)	0 (0.00%)	3 (33.33%)	2 (28.57%)	4 (23.53%)	2 (66.67%)	7 (46.67%)
Arterial thrombi	19 (57.58%)	4 (50.00%)	2 (100%)	7 (77.78%)	4 (57.14%)	13 (76.47%)	2 (66.67%)	11 (73.33%)
Venous thrombi	15 (45.45%)	4 (50.00%)	2 (100%)	7 (77.78%)	2 (28.57%)	10 (58.82%)	2 (66.67%)	3 (20.00%)
Neutrophil infiltration	7 (21.21%)	1 (12.50%)	1 (50.00%)	3 (33.33%)	1 (14.29%)	8 (47.06%)	1 (33.33%)	5 (33.33%)
Bronchial epithelial metaplasia	9 (27.27%)	1 (12.50%)	0 (0.00%)	3 (33.33%)	3 (42.86%)	2 (11.76%)	0 (0.00%)	3 (20.00%)
Bronchial epithelial desquamation	21 (63.64%)	3 (37.50%)	2 (100%)	7 (77.78%)	6 (85.71%)	9 (52.94%)	3 (100%)	11 (73.33%)
Alveolar epithelial metaplasia	13 (39.39%)	2 (25.00%)	1 (50.00%)	5 (55.56%)	3 (42.86%)	3 (17.65%)	1 (33.33%)	6 (40.00%)
Alveolar epithelial desquamation	18 (54.55%)	5 (62.50%)	1 (50.00%)	6 (66.67%)	6 (85.71%)	12 (70.59%)	3 (100%)	12 (80.00%)
Macrophage infiltration of alveoli	16 (48.48%)	5 (62.50%)	2 (100%)	4 (44.44%)	5 (71.43%)	9 (52.94%)	1 (33.33%)	9 (60.00%)
Edematous myxoid stroma	6 (18.18%)	3 (37.50%)	0 (0.00%)	2 (22.22%)	2 (28.57%)	3 (17.65%)	0 (0.00%)	2 (13.33%)
Aspiration	0 (0.00%)	0 (0.00%)	0 (0.00%)	0 (0.00%)	0 (0.00%)	0 (0.00%)	0 (0.00%)	0 (0.00%)
Lymphocytes in alveoli	13 (39.39%)	3 (37.50%)	2 (100%)	4 (44.44%)	4 (57.14%)	6 (35.29%)	2 (66.67%)	7 (46.67%)
Siderophages in bronchi	0 (0.00%)	2 (25.00%)	1 (50.00%)	3 (33.33%)	2 (28.57%)	2 (11.76%)	—	1 (6.67%)

Further evaluation of clinical, laboratory and pathological correlates showed that patients with mean respiration rates of over 21 generally had lower C-reactive protein values (*p* = 0.0316) and showed signs of macrophage infiltration of alveoli more commonly (*p* = 0.022). Patients who were sick for over 14 days spent significantly more time on ventilation (*p* = 0.001), had lower SpO2 levels on admission (*p* = 0.015), commonly presented with granulation tissue (*p* = 0.016), neutrophil pulmonary infiltration (*p* = 0.010) and bronchial epithelial metaplasia (*p* < 0.001) than those who were sick for under 14 days. This suggests that granulation, neutrophil infiltration and epithelial changes are characteristic of disease progression.

Patients who passed away quicker (<7 days of hospitalization) were surprisingly younger (*p* = 0.022), had a higher heart mass (*p* = 0.009), shorter disease duration (*p* < 0.0001), spent less time on ventilation (*p* < 0.0001), had less venous thrombi (*p* = 0.038), less findings of epithelial desquamation (*p* = 0.038), myxoid edema (*p* = 0.045), plasmocytic infiltration (*p* = 0.044). Importantly, these findings did not show any significant variability in terms of comorbidity. Patients aged 70 and under were more often in DAD phase 1–2 (*p* = 0.006), while no other significant age related discrepancies were noted in both comorbidities, clinical and pathological status. Arterial thrombosis was more common in older patients (*p* < 0.0001), and was significantly associated with presence of siderophages in alveoli (*p* = 0.0002), interstitial pulmonary edema (*p* = 0.011), cytopathic effect (*p* = 0.037), pulmonary infarctions (*p* = 0.016) and presence of venous thrombi (*p* < 0.0001). C-reactive protein levels of over 200 were associated with increased macrophageal infiltration of alveoli (*p* = 0.045), reduced myxoid edema (*p* = 0.016) and were more common in patients with ischemic heart disease (*p* = 0.009).

## Discussion

Many publications regarding the safety and protocol for COVID-19 autopsies have been published [9–11,13,14]. Proper ventilation [9,15], full-body protection, breathing filters and other techniques have been recommended to assure isolation of the pathologist [9–11]. In some institutions pathologists follow a special in corpore technique [9], or a pinpoint ultrasound autopsy, while others prefer full or partial evisceration techniques [9–11]. Several institutions do not perform autopsies, favoring full-body post-mortem MRI scanning instead [16]. Such necessary limitations have been widely implemented [17–21]. In our study, we utilized a full-body isolation protocol, which allowed for complete pathological evaluation.

Numerous clinical studies showed the aggravating effects of comorbidities on COVID-19 prognosis [9,15,16,22–28]. Arterial hypertension, coronary artery disease, diabetes mellitus, obesity, malignant neoplasms and chronic pulmonary disease lead to increased lethality risk [6,9,15,16,26,29–35]. In our study, DAD was noted to be the main factor, influencing lethality in patients with existing comorbidities. More so, our revealed the presence of a mixed phase of diffuse alveolar damage with signs of both exudative and proliferative phases in COVID associated pathology, found a discrepancy between the duration of the disease and the phase of DAP (in some cases, the morphological picture of the exudative phase of DAP was observed later than 14 days from the onset of the disease, while in other cases, deposits of fibrin and granulation tissue were present already at 7 days from the onset of the disease). Our findings suggest that granulation, neutrophil infiltration and epithelial changes are characteristic of disease progression. Noting that the subjects of our study all succumb to the disease, it is important to note that these signs may be poop prognostic factors, indicating uncontrolled disease progression.

The limitations of our study involve the sample size: while being one of the largest cohorts reported, the subgroups were often too small for proper statistical evaluation. Furthermore, the lack of data on clinical background of some patients (due to the acute matter of the disease in previously unmonitored patients) posed limitations in analysis of risk factors and individual patterns. A single center analysis poses limitations regarding quality of care, which may vary elsewhere.

Diffuse alveolar damage has several distinct phases: an exudative phase (observed in 1–7 days of illness), followed by a proliferative phase (1–3 weeks since disease manifestation) and a fibrotic phase (if occurs, after 3 weeks). A combination of the proliferative and an exudative phase was seen in 36% of cases in our study, which is consistent with other findings [15,22,36,37]. A prolonging of the exudative phase may be associated with treatment, intervention ventilation and viral load factors. An Italian study showed that among 38 deceased patients hospitalized for 1–23 days, no one was diagnosed with the fibrotic stage of DAD, but rather with the exudative and early proliferative stages of DAD [15].

SARS-CoV-2 replicates mainly within Type II Alveolocytes due to ACE affinity [38,39]. However, Carsana et al. identified viral particles along the plasmalemma and inside the intracytoplasmic vacuoles of Type 1 and 2 Pneumocytes and alveolar macrophages [15]. Bacterial and mycotic pneumonia complicating SARS-CoV-2 infection have been discussed as factors influencing lethality and viral activity [9,15,16]. According to our data, a bacterial (31%) and a bacterial-mycotic (3%) flora was identified in the patients on prolonged artificial ventilation, which partially confirmed this hypothesis.

Vascular complications, including thromboembolic and hemorrhagic, are widely seen in post-mortem COVID-19 autopsies [40–44]. This is due to SARS-CoV-2 associated coagulopathic disorders (generalized thrombotic microangiopathy, vascular endothelial injury) [9,40]. We observed DIC in up to 31% of cases, presented as multiple vascular hemorrhages or thromboses in different locations, pulmonary artery and vein multiple obturating thromboses, including hemorrhagic pulmonary infarcts and extrapulmonary pathology.

According to our data, left ventricular hypertrophy, cardiomyocyte hypertrophy, cellular myocardial degeneration and arterial hypertension-related pathology were the most common findings in the heart, consistent with existing data [16,36,37]. IHC demonstrated that myocardial interstitial portion is mainly presented by macrophages and some amount of CD4^+^ T-cells. CD8^+^ T-cells and CD20^+^ B-cells have not been identified. However, existing studies have not yet identified SARS-CoV-2 viral particles within the myocardium cells [36]. Lymphoplasmacytic infiltration was not identified by many researchers [37], the signs of myocarditis were also absent in our study. Myocardial senile amyloidosis was demonstrated in 1% of cases in our study, and was also noted by other researchers [9].

Several other studies have demonstrated lymphocytic infiltration of hepatic lobules, sinusoid dilatation, partial hepatocyte necrosis, yet were mainly associated with concomitant pathology (e.g., liver cirrhosis) [22,36,37]. Our findings show hepatocyte dystrophy and hepatic degeneration associated with direct COVID-19 associated damage. In our previous work owe showed COVID-19 lymph node damage consists of reduction of lymphoid follicles and expansion of the paracortical zone with reactive plasmacytosis, extrafollicular B-cell activation, sinus histiocytosis, signs of hemophagocytosis, formation of hyaline thrombi, diapedetic hemorrhages, and massive hemorrhages in individual nodes. An immunohistochemical study revealed the predominance of CD4 + T-helpers in the paracortical zone, depletion of cytotoxic CD8 + lymphocytes, an increase in the number of both lymphocytes expressing the PD-1 suppressor protein and activated lymphocytes expressing the CD30 activation antigen. CD123-positive plasmacytoid dendritic cells secreting type 1 interferon were found in a significant amount in the lumen of the sinuses and in the paracortical zone [45].

Damage to the kidneys in COVID-19 presents with arteriolonephrosclerosis along with nephritis, collapse glomerulopathy, tubular epithelium dystrophy with protein exudate, cylinders within enlarged tubules and fibrin thrombi within capillaries [22,36]. Tubular necrosis and renal infarctions, associated with septic conditions, were seen in 2 cases in our study. Splenic enlargement due to sepsis, major follicular reduction, lymphatic infiltration, necrosis and hemorrhages have been shown in our study, consistent with other findings [36]. A B-dependent zone reduction and cytotoxic T-lymphocyte depletion with PD-1 superexpression were characteristic of severe coronaviral infection, which, according to several studies, demonstrates immune response reduction [45]. The paracortical zone showed a significant reactive plasmocytosis with T-helper existence—a morphologic substrate of humoral immunity, which may testify a noneffective humoral response in COVID-19-effected patients with simultaneous T-cell immunity failure.

Our data adds value to analysis of certain potential predictors of disease severity. Respiration rate was shown to be significantly greater in patients with more than one comorbidity, indicating the potential prognostic value of this parameter. Pulmonary findings were non-specific to clinical symptoms, prognosis or disease duration, which highlight the vast unpredictability of the disease, hinting and genetic predisposition to severe forms. Our findings that younger patients passed away sooner may be attributed to the fact that they are admitted at later stages and elderly patients are prioritized in acute care. Patients who do not die early and develop granulations, immune infiltration and epithelial changes are at risk of lethal outcome. Arterial thrombosis was the dominant factor in lethal outcome in the elder patients (over 70), while younger patients did not develop thrombosis, but presented with acute DAD phase 1-2, suggesting it’s main role in lethal outcome of a predisposed cohort.

## Conclusion

The new coronaviral infection pathogenesis and its pathological manifestations are being heavily studied. Due to high contagiousness and low autopsy rate worldwide, too few autopsies of the coronavirus-affected patients are performed. As such, our study provides substantial insight into the macro- and micropathological aspects of COVID-19 damage. Our results show high prevalence of diffuse alveolar damage, as well as immune compromise and polyorgan failure in deceased patients.

## Data Availability

The raw data supporting the conclusion of this article will be made available by the authors, without undue reservation.

## References

[1] Reicher S Stott C. On order and disorder during the COVID‐19 pandemic. Br J Soc Psychol (2020) 59(3):694–702. 10.1111/bjso.12398 32609398 PMC7361727

[2] Dong E Du H Gardner L. An interactive web-based dashboard to track COVID-19 in real time. Lancet Inf Dis 20(5):533–4. 10.1016/S1473-3099(20)30120-1 PMC7159018 32087114

[3] Kobak D. Excess mortality reveals Covid's true toll in Russia. Significance (2021) 18(1):16–9. 10.1111/1740-9713.01486 33821160 PMC8013319

[4] Pomeranz JL Schwid AR. Governmental actions to address COVID-19 misinformation. J Public Health Pol (2021) 42:201–10. 10.1057/s41271-020-00270-x PMC7841970 33510401

[5] Partinen M Bjorvatn B Holzinger B Chung F Penzel T Espie C. A ICOSS collaboration group. Sleep and circadian problems during the coronavirus disease 2019 (COVID‐19) pandemic: the International COVID‐19 Sleep Study (ICOSS). J Sleep Res (2021) 30(1):e13206. 10.1111/jsr.13206 33179820

[6] Riiser K Helseth S Haraldstad K Torbjørnsen A Richardsen K. R. Adolescents’ health literacy, health protective measures, and health-related quality of life during the Covid-19 pandemic. PloS one (2020) 15(8):e0238161. 10.1371/journal.pone.0238161 32857806 PMC7454983

[7] Liu C. H Stevens C Conrad R. C Hahm H. C. Evidence for elevated psychiatric distress, poor sleep, and quality of life concerns during the COVID-19 pandemic among U.S. young adults with suspected and reported psychiatric diagnoses. Psychiatry Res (2020) 292:113345. 10.1016/j.psychres.2020.113345 32745794 PMC7387248

[8] Calabrese F Fortarezza F Fortarezza F Hofman P Kern I Panizo A Lunardi F. Diffuse Idiopathic Pulmonary Neuroendocrine Cell Hyperplasia (DIPNECH). Virchows archiv (2020) 1–7. 10.1007/978-3-319-28845-1_5073-1

[9] Batah S. S Fabro A. T. Pulmonary pathology of ARDS in COVID-19: A pathological review for clinicians. Respir Med (2020) 106239. 33246294 10.1016/j.rmed.2020.106239 PMC7674971

[10] Barton L. M Duval E. J Stroberg E Ghosh S Mukhopadhyay S. COVID-19 Autopsies, Oklahoma, USA. Am J Clin Pathol (2020) 153(6):725–33. 10.1093/ajcp/aqaa062 32275742 PMC7184436

[11] Hanley B Lucas S. B Youd E Swift B Osborn M. Autopsy in suspected COVID-19 cases. J Clin Pathol (2020) 73:239–42. 10.1136/jclinpath-2020-206522 32198191

[12] Mauad T Hajjar L. A Callegari G. D da Silva L. F. F Schout D Galas F. R. B. G Lung pathology in fatal novel human influenza A (H1N1) infection. Am J Respir Crit Care Med (2010) 181(1):72–9. 10.1164/rccm.200909-1420oc 19875682

[13] Centers for Disease Control and Prevention. Collection and Submission of Postmortem Specimens from Deceased Persons with Known or Suspected COVID-19, March 2020 (Interim Guidance). Available from: www.cdc.gov/coronavirus/2019-ncov/hcp/guidance-post mortem-specimens.html (Accessed May, 2020).

[14] College of American Pathologists (CAP). Amended COVID-19 autopsy guideline statement from the CAP autopsy committee (2020). Available from: https://covid19.who.int/ (Accessed May, 2020).

[15] Carsana L Sonzogni A Nasr A Rossi RS Pellegrinelli A Zerbi P Pulmonary post-mortem findings in a series of COVID-19 cases from northern Italy: a two-centre descriptive study. Lancet Infect Dis (2020) 20(20):1135–40. 10.1016/S1473-3099(20)30434-5 32526193 PMC7279758

[16] Wichmann D Sperhake JP Lütgehetmann M Steurer S Edler C Heinemann A Autopsy Findings and Venous Thromboembolism in Patients With COVID-19 [published online ahead of print, 2020 May 6]. Ann Intern Med (2020) M20–2003. 10.7326/M20-2003 PMC7240772 32374815

[17] Centers for Disease Control and Prevention (CDC). Interim laboratory biosafety guidelines for handling and processing specimens associated with coronavirus disease 2019 (COVID-19) (2020). https://www.cdc.gov/coronavirus/2019-nCoV/lab/lab-biosafetyguidelines.html (Accessed May, 2020).

[18] World Health Organization. Laboratory biosafety guidance related to the novel coronavirus (2019-nCoV): interim guidance (2020). https://www.who.int/publications-detail/laboratory-biosafetyguidance-related-to-coronavirus-disease-2019-(covid-19 (Accessed May, 2020).

[19] Iwen P. C Stiles K. L Pentella M. A. Safety Considerations in the Laboratory Testing of Specimens Suspected or Known to Contain the Severe Acute Respiratory Syndrome Coronavirus 2 (SARS-CoV-2). Lab Med (2020) 51:239–42. 10.1093/labmed/lmaa018 32275309 PMC7184424

[20] Barbareschi M Ascoli V Bonoldi E Cavazza A Colombari R Cozzi I Biosafety in surgical pathology in the era of SARS-Cov2 pandemia. A statement of the Italian Society of Surgical Pathology and Cytology. Pathologica (2020) 12(2):59–63. 10.32074/1591-951X-14-20 PMC7931564 32324726

[21] Henwood A. F. Coronavirus disinfection in histopathology. J Histotechnology (2020) 43:102–4. 10.1080/01478885.2020 32116147

[22] Duarte-Neto AN Aparecida de Almeida Monteiro R Ferraz da Silva LF Costa Malheiros DM. A de Oliveira E. P Filho JT Pulmonary and systemic involvement of COVID-19 assessed by ultrasound-guided minimally invasive autopsy. Histopathology 77(2):186–97. 10.1111/his.14160 PMC7280721 32443177

[23] Zhang H Zhou P Wei Y Yue H Hu M Zhang S Histopathologic Changes and SARS-CoV-2 Immunostaining in the Lung of a Patient With COVID-19. Ann Intern Med (2020) 172(9):629–32. 32163542 10.7326/M20-0533 PMC7081173

[24] Tang N Li D Wang X Sun Z. Abnormal coagulation parameters are associated with poor prognosis in patients with novel coronavirus pneumonia. J Thromb Haemost (2020) 18(4):844–7. 10.1111/jth.14768 32073213 PMC7166509

[25] Tang N Bai H Chen X Gong J Li D Sun Z. Anticoagulant treatment is associated with decreased mortality in severe coronavirus disease 2019 patients with coagulopathy. J Thromb Haemost (2020) 18(5):1094–9. 10.1111/jth.14817 32220112 PMC9906401

[26] Zhou F Yu T Du R Fan G Liu Y Liu Z Clinical course and risk factors for mortality of adult inpatients т with COVID-19 in Wuhan, China: a retrospective cohort study [published correction appears in Lancet. 2020;395(10229):1038. 10.1016/s0140-6736(20)30566-3 PMC7270627 32171076

[27] Wang Y Lu X Li Y Chen H Chen T Su N Clinical Course and Outcomes of 344 Intensive Care Patients with COVID-19. Am J Respir Crit Care Med (2020) 201(11):1430–4. 10.1164/rccm.202003-0736le 32267160 PMC7258632

[28] Du Y Tu L Zhu P Mu M Wang R Yang P Clinical Features of 85 Fatal Cases of COVID-19 from Wuhan. A Retrospective Observational Study. Am J Respir Crit Care Med (2020) 201(11):1372–9. 10.1164/rccm.202003-0543oc 32242738 PMC7258652

[29] Guan W-j. Ni Z-y. Hu Y Liang W-h. Ou C-q. He J-x. Clinical Characteristics of Coronavirus Disease 2019 in China. N Engl J Med (2020) 382:1708–20. 10.1056/NEJMoa2002032 32109013 PMC7092819

[30] Wu C Chen X Cai Y Xia J Zhou X Xu S Risk factors associated with acute respiratory distress syndrome and death in patients with coronavirus disease 2019 pneumonia inWuhan, China. JAMAInternMed (2020) 180(7):934–43. 10.1001/jamainternmed.2020.0994 PMC7070509 32167524

[31] Zayratyants OV Samsonova MV Mikhaleva LM Chernyaev AL Mishnev OD Krupnov NM Moscow, GBU «NIIOOZM DZM»,.– (2020). p. 142. 33760431

[32] Xu Z Shi L Wang Y Zhang J Huang L Zhang C Pathological findings of COVID-19 associated with acute respiratory distress syndrome. Lancet Respir Med (2020) 8:420–2. 10.1016/S2213-2600(20)30076-X 32085846 PMC7164771

[33] Fox S. E Akmatbekov A Harbert J. L Li G Brown J. Q Heide R. S. V. Pulmonary and Cardiac Pathology in Covid-19: The First Autopsy Series from New Orleans. Lancet Respir Med. 8(7):681–6. 10.1101/2020.04.06.20050575 PMC7255143 32473124

[34] Dolhnikoff M Duarte-Neto AN de Almeida Monteiro RA da Silva LFF de Oliveira EP Nascimento Saldiva PH Pathological evidence of pulmonary thrombotic phenomena in severe COVID-19. J Thromb Haemost (2020) 18(6):1517–9. 32294295 10.1111/jth.14844 PMC7262093

[35] Lillicrap D. Disseminated intravascular coagulation in patients with 2019‐nCoV pneumonia. J Thromb Haemost (2020) 18(4):786–7. 10.1111/jth.14781 32212240 PMC7166410

[36] Yao XH Li TY He ZC Ping YF Liu HW Yu SC [A pathological report of three COVID-19 cases by minimal invasive autopsies]. Zhonghua Bing Li Xue Za Zhi (2020) 49(0):411–7. 10.3760/cma.j.cn112151-20200312-00193 32172546

[37] Tian S Xiong Y Liu H Niu L. Pathological study of the 2019 novel coronavirus disease (COVID-19) through post-mortem core biopsies. Mod Pathol (2020) 33:1007–1014. 32291399 10.1038/s41379-020-0536-x PMC7156231

[38] Mason R. J. Pathogenesis of COVID-19 from a cell biology perspective. Eur Respir J (2020) 55(4):2000607. Published 2020 Apr 16. 10.1183/13993003.00607-2020 32269085 PMC7144260

[39] Lukassen S Chua R. L Trefzer T Kahn N. C Schneider M. A Muley T SARS ‐CoV‐2 receptor ACE 2 and TMPRSS 2 are primarily expressed in bronchial transient secretory cells. EMBO J (2020) 39:e105114. 10.15252/embj.20105114 32246845 PMC7232010

[40] Ackermann M Verleden S. E Kuehnel M Haverich A Welte T Laenger F Pulmonary Vascular Endothelialitis, Thrombosis, and Angiogenesis in Covid-19. N Engl J Med (2020) 383(2):120–8. 10.1056/nejmoa2015432 32437596 PMC7412750

[41] Kollias A Kyriakoulis K. G Dimakakos E Poulakou G Stergiou G. S Syrigos K. Thromboembolic risk and anticoagulant therapy in COVID‐19 patients: emerging evidence and call for action. Br J Haematol (2020) 189(5):846–7. 10.1111/bjh.16727 32304577 PMC7264537

[42] Grasselli G Zangrillo A Zanella A Antonelli M Cabrini L Castelli A Baseline Characteristics and Outcomes of 1591 Patients Infected With SARS-CoV-2 Admitted to ICUs of the Lombardy Region, Italy [published online ahead of print, 2020 Apr 6]. JAMA (2020) 323(16):1574–81. 10.1001/jama.2020.4031 32250385 PMC7136855

[43] Klok F. A Kruip M. J. H. A van der Meer N. J. M Arbous M. S Gommers D. A. M. P. J Kant K. M Incidence of thrombotic complications in critically ill ICU patients with COVID-19. Thromb Res (2020) 191:145–7. 10.1016/j.thromres.2020.04.013 32291094 PMC7146714

[44] Oudkerk M Büller HR Kuijpers D Van Es N Oudkerk S. F McLoud T Diagnosis, Prevention, and Treatment of Thromboembolic Complications in COVID-19. Radiology (2020) 201629. Report of the National Institute for Public Health of the Netherlands [published online ahead of print, 2020 Apr 23]. 10.1148/radiol.2020201629 PMC7233406 32324101

[45] Kovrigina A. M Shalamova EA Shalamova E. A Berezovskiy Y. S Kalinin D. V Gretsov E. M Pathomorphological and immunohistochemical features of lymph nodes in COVID-19 patients (autopsy study). Clin Exp Morphol (2020) 9(Dec. 2020):12–23. 10.31088/CEM2020.9.4.12-23

